# Capturing subjective experiences of atypical depression: qualitative investigation of perceived aetiological factors and gender influences

**DOI:** 10.1192/bjo.2025.10924

**Published:** 2025-12-17

**Authors:** Ruxandra Ioana Toma, Lauren Blunstone, Mario Juruena, Susannah Pick

**Affiliations:** Department of Psychological Medicine, Institute of Psychiatry, Psychology & Neuroscience, King’s College London, London, UK

**Keywords:** Atypical depression, aetiology, gender, trauma, qualitative

## Abstract

**Background:**

Subjective perspectives on aetiological factors in atypical depression have not been previously explored from the viewpoint of those with lived experience.

**Aims:**

This study aimed to explore individuals’ subjective experiences and explanations of atypical depression, and to examine whether perceived gender-specific influences might contribute to the observed gender disparity in atypical depression prevalence.

**Method:**

Semi-structured, one-to-one interviews were conducted online with 16 individuals. Data were analysed using thematic analysis, employing an inductive approach and interpreted within a constructionist framework. Data coding was conducted using NVivo.

**Results:**

Key themes centred on the prevalence of comorbid conditions and how they affected atypical depression presentation; how trauma was seen as both a causal factor and catalyst; the subjective impact of gender identity and roles; how environmental factors seemed to affect atypical depression onset and presentation; the difficulties experienced with atypical depression symptom variability in daily life; and reported coping behaviours.

**Conclusions:**

These findings highlight how individuals with atypical depression believed onset to be linked to experiences of trauma and comorbidity, in addition to ongoing influences of varied environmental factors. The variability of atypical depression symptoms in both the short and long term appears to be a core challenge in this subgroup. The gender disparity of atypical depression is also explored through the lens of lived experience and gender identity. Future research would benefit from exploring further these potential contributing factors, to provide a better understanding of their complex influences on atypical depression onset and maintenance.

Major depressive disorder (MDD) is defined as involving at least 2 weeks of persistent low mood and/or loss of interest, accompanied by cognitive and somatic symptoms such as fatigue, worthlessness, impaired concentration and decreased appetite.^1^ While MDD is associated with heightened functional impairment and high rates of comorbidity with other psychiatric disorders,^1,2^ its heterogeneity has been increasingly recognised with the inclusion of subtypes and specifiers in diagnostic frameworks.^1^ Proposed subtypes include those with anxious distress, mixed symptoms and psychotic, melancholic or atypical features (atypical depression), which are seemingly associated with diverse ages of onset, comorbidity patterns and possible causal contributions.^3,4^ An all-encompassing MDD diagnosis, therefore, risks homogenising a multifaceted clinical entity,^5^ potentially resulting in imprecise or ineffective treatment recommendations. Investigating subtypes of depression, including potential differences in aetiology, mechanisms and treatments response profiles, is of critical importance for improving the precision of diagnosis and intervention in MDD.

Around 15% of MDD patients present with atypical features,^2^ which are twice as common in women than in men.^6^ Atypical depression has the primary feature of mood reactivity, alongside at least two of hypersomnia, increased appetite/weight, ‘leaden paralysis’ and chronic rejection sensitivity.^1^ Atypical depression was first introduced in the fourth edition of the Diagnostic and Statistical Manual of Mental Disorders (DSM-IV), and has been supported subsequently as a subtype with a unique psychological profile and possibly differing mechanisms from other subtypes of depression.^7–11^ Atypical depression appears to be associated with a younger age of onset and greater chronicity compared with other subtypes,^8^ in addition to increased rates of comorbid psychiatric diagnoses, particularly anxiety disorders and bipolar spectrum disorders,^7,8^ as well as adverse cardiometabolic profiles.^9^ Individuals with atypical depression are more likely to engage in multiple high-risk behaviours than the general population, such as heavy smoking and alcohol misuse, alongside high obesity and diabetes rates,^9^ possibly contributing to an elevated risk of premature mortality.^10^ Despite the noted increase in risk and comorbidity associated with atypical depression, the aetiology and clinical presentation of this subtype has remained understudied, and available findings relating to its potential causes and features have been inconsistent.

From a psychosocial perspective, limited evidence indicates that atypical depression may be more strongly associated with adverse childhood experiences (e.g. abuse) compared with melancholic depression.^10–14^ More broadly, higher trauma exposure is related to earlier onset, depression chronicity and increased comorbidity in MDD, which are also characteristic of atypical depression.^11–14^ Increased trauma exposure has been reported both before and following atypical depression onset.^12^ These findings might be related to the observed positive association between hypothalamic–pituitary–adrenal (HPA) axis downregulation and atypical depression.^12^ Hypoactivity in the HPA axis characterises both trauma victims and atypical depression, with similar clinical presentations of increased emotional reactivity and decreased stress tolerance.^14^ It is possible, therefore, that trauma may play a unique role in atypical depression, inciting onset through gene and environmental vulnerabilities. At the same time, symptom presentation in atypical depression may also increase the likelihood of experiencing further interpersonal trauma: for instance, heightened interpersonal rejection sensitivity reduces the ability to manage negative relationship dynamics.^14–16^

Lower self-esteem may also be of greater importance in atypical depression compared with melancholic depression.^14^ Reduced self-esteem is not a specifier for either atypical depression or melancholic depression in DSM-V-TR; however, low self-esteem has been linked to heightened sensitivity to rejection,^14–17^ a hallmark feature of atypical depression. Additionally, weight gain is associated with lowered self-esteem, subsequently linked to reduced self-worth and social rejection.^15^ This is particularly the case for women who experience greater body dissatisfaction.^16^ Therefore, there may be interactions among appetite fluctuation, societal standards and self-esteem in atypical depression, potentially contributing to the increased female prevalence.^17^

Due to the lack of qualitative work in atypical depression, there is no consensus on the effect of gender roles and discourse on its perceived causes and prevalence.^18,19^ Gender is defined as a sociopolitical and cultural viewpoint of what it ‘means’ to be a woman or a man.^20^ From a social constructionist perspective, ‘men’ and ‘women’ are fluid constructs derived from broader societal structures.^21^ This study therefore views gender as a societal construct interacting with other vulnerability factors that may, together, contribute to atypical depression onset.^17,19^ From a sociocultural perspective, this influence is exerted through gender roles – socially constructed expectations that determine behaviours and values deemed appropriate for women and men.^18^ These gender-specific values, developed through socialisation, subsequently influence identity formation and emotional expression.^21^

Gender role adherence has been linked to individual perception of mental health. In a study analysing gender role conflict and self-stigmatising perceptions of MDD, endorsement of traditionally masculine gender roles increased rumination and self-stigmatising beliefs in men.^22^ This was supported by similar studies which have found that rigid conformity to gender norms correlated with increased mental health issues.^23^ This relationship remains complex, because a stressor’s effect is often regulated by individual meaning and relationship to the self-complex.^24^ For example, in a qualitative study regarding gendered expressions of depression, gender role socialisation and socioeconomic background had similar influences but lower socioeconomic status increased hopelessness in men and women regardless of gender role adherence.^25^ Therefore, the effects of gender on mental health must be considered alongside other factors. However, it has not been determined how these interactions may affect atypical depression development or onset. Gender interacts with many biopsychosocial factors, including hormones, genetic vulnerabilities and negative life events.^20^ Therefore, additional research is required to understand this interaction on an individual level.

To our knowledge, no previous qualitative studies have explored lived experiences of, and beliefs about, atypical depression and its aetiology. Existing qualitative literature has highlighted the role of individual beliefs in MDD aetiology and treatment management, with findings suggesting that current life stressors and lifetime trauma are often attributed to MDD aetiology, with less focus on biological vulnerability.^22,23^ A study with Swedish primary care patients found that patients’ explanatory models influenced help-seeking behaviours, treatment and medication adherence. Notably, individuals who attributed less importance to biological factors in their explanatory model of MDD were also less likely to consent to pharmacological treatment for MDD.^23^ Furthermore, a qualitative meta-review highlighted that patients’ interpretative frameworks were influenced by sociocultural values, with individuals of ethnic and cultural minorities often experiencing a lack of reciprocal understanding within healthcare systems, which hindered their engagement with interventions.^22^

Exploring patients’ beliefs about, and experiences of, atypical depression is essential for deriving insights into interactions among individual interpretations, beliefs and aetiological influences.^23^ Furthermore, individual beliefs and interpretations of psychological symptoms have significant clinical implications, affecting help-seeking behaviours, cognition and confidence in, and compliance with, pharmacological treatment.^19^ Therefore, it is essential to explore the subjective experiences of those with first-hand atypical depression experience.

Through the lens of social constructionism,^21^ this study aimed to understand individuals’ subjective experiences and explanations of atypical depression. We also aimed to investigate whether there are gender-specific influences that may contribute to the perceived causes of atypical depression, potentially informing a better understanding of the gender disparity in this subgroup. We sought to examine the following research questions: (a) What are the biopsychosocial aetiological factors that people with atypical depression perceive to be associated with atypical depression? (b) Do people with atypical depression perceive gender-related factors to be relevant to their illness?

## Method

The authors assert that all procedures contributing to this work comply with the ethical standards of the relevant national and institutional committees on human experimentation, and with the Helsinki Declaration of 1975 as revised in 2013. Ethical approval for all procedures involving human subjects was approved by the King’s College London health faculties Research Ethics Committee (no. HR/DP-23/24-40841). Written consent was obtained from all participants for both participation and publication of anonymised data.

### Design

Semi-structured qualitative interviews were chosen to engage participants’ subjective experiences of atypical depression. Participant experience is acknowledged as being influenced by sociocultural contexts. This study adopted a social constructionist framework, underpinned by the view that knowledge is created through social interaction rather than existing independently. This reflects an epistemological stance that individuals mentally construct their perspective through social processes within both a subjective and objective reality.^21^ The design was also informed by role theory and gender role theory, which conceptualise behaviour as emerging from socially defined roles and expectations.^24^ Our analysis aimed to construct reflexively individual beliefs within the participant’s social world, rather than portray them as a passive medium.^24^ Consequently, this study was designed to highlight connections between wider sociocultural norms and participants’ inner discourse.^20,21^

### Participants and recruitment

Recruitment for this study took place between February and May 2024. Participants were recruited via advertisements on social media platforms (e.g. Twitter, Facebook) and the King’s College London Research Volunteer circular bulletin. All participants took part in a quantitative sub-study first (to be reported elsewhere), which involved an initial set of online questionnaires administered separately. Based on scores on the Inventory of Depressive Symptomatology (IDS-SR^26^) questionnaire, participants who met the criteria for current or previous atypical depression were invited to participate in this qualitative study. Participants were excluded from the study if their age was not between 18 and 65 years: this upper and lower limit is intended to maintain homogeneity in the sample, because aetiological factors might differ between children and older adults. Exclusion criteria also included suffering from severe mental illness or physical conditions, which could have compromised completion of the study. Additionally, if invalid response patterns were identified, participants were excluded retrospectively.

Twenty participants were interviewed. Four participants were excluded retrospectively due to inconsistent response patterns between the questionnaires and interview. Sixteen eligible participants were included in this analysis: ten women, two non-binary individuals and four men. None of the participants requested withdrawal from the study, or to withdraw their data. Therefore, all 16 eligible participants were included in the analysis. Demographic characteristics for all participants can be found in Table 1.

**Table 1:** Demographic information

Participant code	Age (years)	Gender	Sexuality	Ethnicity	Occupational status	Education	Comorbidity
P1	20s	F	Other	Indian	Student	A-levels	Hypothyroidism, panic disorder
P2	40s	M	Heterosexual	Pakistani	Professional	Postgraduate degree	None disclosed
P3	20s	M	Heterosexual	Black	Professional	Undergraduate degree	Anxiety (self-described)
P4	20s	F	Heterosexual	Asian	Professional	Postgraduate degree	None disclosed
P5	20s	NB	Other	White	Student	A-levels	None disclosed
P6	40s	F	Heterosexual	White	Professional	Postgraduate degree	None disclosed
P7	30s	M	Heterosexual	White	Professional	Doctoral degree	Anxiety (self-described)
P8	30s	F	Other	White	Administration	Postgraduate degree	None disclosed
P9	20s	F	Bisexual	White	Professional	Undergraduate degree	Generalised anxiety disorder
P10	20s	M	Heterosexual	Indian	Student	Undergraduate degree	None disclosed
P11	20s	GNC	Other	White	Student	A-levels	None disclosed
P12	20s	F	Undisclosed	White	Professional	Undisclosed	Anxiety (self-described)
P13	20s	F	Heterosexual	Undisclosed	Placement student	A-levels	Anxiety (self-described
P14	20s	F	Other	White	Professional	Undergraduate degree	Anxiety (self-described)
P15	30s	M	Heterosexual	African	Professional	Postgraduate degree	None disclosed
P16	30s	M	Gay	White	Unemployed	A-levels	Fibromyalgia. panic disorder, agoraphobia

F, female; M, male; NB, non-binary; GNC, gender non-conforming.

### Materials

Semi-structured interviews used a pre-established guide (see supplementary materials available at https://doi.org/10.1192/bjo.2025.10924). This contained six questions, each with two to three associated prompts to further engage or clarify participants’ answers. Participants were contextually probed, as required, to maximise clarity of understanding.

The interview guide included two parts. The first part contained questions regarding the participant’s subjective experiences of atypical depression, specifically inviting them to share their perspectives about what might have contributed to their depression. The second half of the interview focused on their perspective on the interaction between their gender identity and atypical depression. Prompts also included clarifications about whether other factors, such as culture or socioeconomic status, had played a role in their experiences.

The interview guide drew on existing qualitative research on lived experience in MDD^25,27^ and was developed with input from individuals with personal experience of mental health symptoms, including atypical depression.

Interviews were recorded and transcribed through the Microsoft Teams automatic transcription function, and were subsequently edited manually for accuracy by the first author (R.I.T.).

### Procedure

Recruitment was conducted online. The recruitment advertisement invited participants to contact the research team through email. Following initial contact, preliminary screening questions established the basic eligibility criteria. Participants were sent an information sheet and consent form if potentially eligible. Once any questions had been answered and the consent form was signed, participants were screened using an online version of IDS-SR. Participants next completed a more extensive set of online questionnaires (to be reported elsewhere). A subsample of participants with atypical depression were invited to the interview. Selection for the interviews was based on participants’ availability and willingness to participate, alongside demographic characteristics to ensure diverse representation in terms of age and gender. Participants were then re-sent the information sheet to review and a separate consent form specific to the qualitative part of the study.

All interviews were conducted remotely by R.I.T. using Microsoft Teams. Any identifiable information shared by participants was removed from transcripts with manual edits during the initial stage of checking and editing transcripts, and marked accordingly by square brackets. The individual interviews lasted between 30 and 60 min. A standard 5 min break was provided, with the option of requesting additional breaks for any reason. Participants were reimbursed with a 20 GBP shopping voucher for their time.

Due to the online nature of this study and early detection of some fraudulent respondents (e.g. clearly inconsistent or invalid responses), the research team were vigilant in identifying potential invalid responses across both the quantitative (questionnaire) and qualitative (interview) elements of the study. Due to significant inconsistencies between online questionnaire responses and interview transcripts in some instances, identified by the interviewer/research team and/or the research supervisor (S.P.), four respondents were excluded retrospectively from the qualitative study. These inconsistencies raised concerns about data validity and the possibility of duplicate participation and, as such, the affected cases were omitted from the final analysis.

### Data analysis

Reflexive thematic analysis was used for data analysis, employing a constructionist paradigm.^28,29^ Participants’ experiences were coded as lived realities within wider social contexts and the aforementioned philosophical stance.^20,24^ Respectively, their narratives were coded as being embedded within sociocultural discourse and structural influences. The analysis thus aimed to highlight participants’ accounts as both reflecting, and being constituted through, social discourse and role expectations. This involved an inductive process where codes were generated from the data per se, rather than being predetermined by theoretical constructs. Both semantic and latent codes were developed to capture surface meanings and deeper, socially embedded patterns.

Data analysis was coded by R.I.T. using NVivo version 14 for macOS (Lumivero, Denver, Colorado, USA; https://lumivero.com/products/nvivo/). Familiarisation with the data-set began by manually editing transcripts while listening to audio recordings post-interview. This was followed by listening to a separate playback of the interview and re-reading each transcript numerous times. Following satisfaction that transcripts were accurate, notes and observations were documented in a reflexivity journal. After this step, initial coding commenced using NVivo. This included repeated iterations of coding and further familiarisation; coding evolution was tracked in a reflexivity journal in OneNote (2016). Early coding iterations focused on semantic coding and exploring data patterns and common experiences by participants – later revisions focused on latent coding as diversity in participants and data patterns became apparent. This was also tracked using ‘coding stripe’ features in NVivo. When developing themes, ‘mind’ and ‘concept’ mapping features were used to visualise and explore relationships between code patterns to develop initial themes. After reviewing candidate themes and revisiting interview data, as well as reflexivity notes and maps, six themes were developed and categorised in relation to the interview data and research questions.

### Positionality

The lead analyst and first author (R.I.T.) identifies as a cisgender woman with a longstanding academic interest in mental health, gender, discourse and social constructionist theories. These experiences informed her epistemological stance that aims to acknowledge and highlight participant experience throughout the research process. This analysis was conducted reflexively via regular discussions with the senior author (S.P.), to provide an additional interpretative lens. S.P. is a female academic psychologist with expertise in quantitative and qualitative mental health research, including ongoing studies on aetiological factors and potential mechanisms in MDD subtypes. Additionally, by keeping a thorough reflexivity journal, the lead analyst’s evolving thoughts and decisions were reviewed consistently to assure that these aligned with both participant experience and the study framework. Thus, both the first and senior authors actively shaped the analytic process presented in the following sections. Our coding scheme has been reviewed and commented upon by individuals with personal experience of atypical depression.

## Results

Six themes were developed through reflexive engagement with the data, encompassing (a) the impact of comorbid conditions on atypical depression; (b) the role of trauma; (c) the impact of gender identity and roles; (d) environmental factors; (e) variability in atypical depression symptoms; and (f) coping behaviours.

### Comorbidity as a lens for atypical depression: the impact of comorbid conditions on atypical depression

This theme captured the role of comorbidities in both onset and perpetuation of atypical depression. Comorbidities were experienced by half of the sample – most commonly anxiety disorders, which were described as interdependent with atypical depression symptomatology:

‘I think that for me [depression and anxiety] (…) either happen at the same time or they happen one after the other (…) that probably contributed to it’. Participant 1 (P1)

This relationship seemed to affect atypical depression progression and presentation. Participants described identifying anxiety before depression; in fact, the more anxious participants felt, the more debilitating they discussed their atypical depression to be. Experiencing anxiety alongside atypical depression led to a vicious cycle of rumination which, alongside other stressors, triggered the onset of a new episode (‘…my depressive symptoms also bring out a lot of anxiety’, P11).

Most participants defined their experience with atypical depression as deeply intertwined with other conditions, where anxiety was not only contributing to, but also defining, their atypical depression symptoms. This often also reshaped their self-concept and perception of their diagnosis:

‘I think anxiety is a major contributing factor to my depression (…) I do wonder its relationship to, you know, some of those episodes, whether that maintains them, making it more difficult (…) I’m quite difficult to treat because of that’. P16

For participants describing physical comorbidities, atypical depression was intrinsically linked with bodily sensations. Specifically, their atypical depression was fundamentally experienced through the bodily sensations and limitations imposed by physical conditions. Participants focused their descriptions around the long-term effects of chronic pain and fatigue: ‘Widespread pain in the body has potentially made it worse, or been also a trigger’ (P16). This also affected narrative understanding of atypical depression:

‘It’s always been with me since then (…) I’m just going to have to learn to adapt my life, I guess. I can’t see myself getting “cured” per se, it’s just something that I know that I need to manage’. P14

For these participants, discussing their atypical depression involved the intertwined experience of comorbidity, with each reinforcing one another. Therefore, a significant proportion of participants constructed their understanding of atypical depression alongside that of their comorbidity. This became a key feature through which atypical depression aetiology was interpreted and experienced.

### Trauma and the self: the role of trauma

Most interviews revealed experiences of one or more traumatic life events. Participants talked of these events as having significantly altered their relationship with themselves and those around them. Particularly, participants with multiple or early-onset trauma described prematurely emerging depressive symptoms. Early-onset trauma represented more than an aetiological factor, rather highlighting a core aspect of their selfhood:

‘All of that [trauma] has made me the person that I am today’. P6

Trauma experienced in later life was also discussed as contributing to atypical depression onset. Interpersonal relationship breakdowns were commonly relayed as a catalyst for an atypical depression episode, following heightened anxiety and avoidance of confrontation and communication. Much of participants’ experiences around interpersonal relationship breakdowns centred on dysfunctional relationship dynamics. Specifically, participants discussed a cycle where their trauma was caused by, and maintained in, unhealthy relationships:

‘The relationship was not good for me mentally at all. But I was stuck in it, and I couldn’t see myself leaving it. I think that stemmed from the low confidence and not feeling like I could do anything, because I felt, “if I leave him then I’m going to be alone forever”’. P14

Abuse and trauma fundamentally changed the way participants related to, and understood, the narrative course of their atypical depression. Changes to their self-concept were subsequently relayed as responsible for depression onset. Their trauma either triggered or exacerbated pre-existing vulnerabilities (*‘*…even after I had grieved, it didn’t get better down the line’, P3).

Most participants had family histories of struggles with mental health and/or substance abuse issues. As such, some trauma processing and coping behaviours were linked to childhood environments:

‘I think having to manage my mum’s mental health made me feel really hopeless. Nothing I would try to help would work’. P11

Previous family history of bipolar or MDD diagnoses was discussed by several participants, with an awareness of both direct and indirect biological factors. Participants described a unique interaction between trauma, familial risk and atypical depression onset, which had a substantial psychological impact. Overall, participants perceived trauma as playing a unique role in atypical depression through a complex interplay of genetic and environmental interactions:

*‘*My mum has depression. She hasn’t had it treated appropriately, so I think it affects her a lot more than it should (…) I wonder how much of this I’ve learnt from her’. P9

### Gender, gender roles and biological sex: considering impact

Gender was a divided topic among participants. Negative effects transpiring from rigid gender-specific factors were mainly discussed in an indirect, existential sense rather than lived experience:

*‘*(…) women are pushed into specific roles (…) be a caregiver and all of that. But then I couldn’t say that if I was a man, I wouldn’t be depressed’. P8

Gender identity was similarly portrayed as an abstract factor mediated by culture and environment. Thus, its effects on atypical depression were subsequently dependent on how participants individually related to, and valued, this interaction:

‘I do come from a culture where women are considered inferior to men (…) But my family has been very open’. P1

However, for a subsection of participants, gender played a significant role in their atypical depression. Men unanimously described a pressure to adhere to traditional masculine presentations:

‘In society, on TV, males … are the ones who have to hold everything together’. P2

All male participants, regardless of social status, felt strong societal pressure to uphold traditional masculine appearances, leading to masking symptoms and worsening feelings of low self-worth:

‘They say the same things. “Oh, you don’t have a routine now.” “That must be why you’re depressed” (…) “I just can’t pull myself together!” Those are not the words I feel. They’re the words of what other people say’. P16

Female participants expressed concern about maintaining societal expectations of femininity, particularly regarding physical appearance during depressive episodes. They also recognised negative societal stigma surrounding menstruation (‘There’s a stereotype that women are more sensitive and overdramatic’, P4), described falling short of gender expectations as diminishing self-worth (‘I’m not going with the expectations of my gender… that exacerbates being self-critical’, P8). Menstruation was also discussed as influencing symptoms both somatically and emotionally; for some with anxiety comorbidities, it acted as a ‘catalyst’ that intensified rather than triggered atypical depression symptoms:

‘I have painful cramps… I feel like that does play a part [in my symptoms]’. P4

Although few participants had caregiving responsibilities, these were unanimously described in gender-specific terms. For the minority of participants that felt they fell short of traditional expectations of their respective caregiving role, this conflict was linked to decreased low self-esteem (‘You feel like a failure to an extent, as a father … you’re there to protect, provide’, P2).

However, many participants discussed a gendered aspect to caregiving, which they were aware would impact them at some point in time (‘being a man entails that you have to think about how to start a life of your own’, P15).

Variability in this theme is also recognised through the lens of two participants who identified as non-binary. These individuals expressed previous gender identity conflict as having contributed to feelings of isolation. However, a gender-diverse identity provided new-found freedom around both symptom presentation and help-seeking behaviour, thus not being a current influence on atypical depression:

‘I don’t feel pressure to not express anger (…) I got such a pushback for feeling angry when I identified as a female, that would make me more upset that I couldn’t express my genuine emotions. But now I don’t feel that push back. I don’t even feel like I have to be angry any more’. P5

### Social determinants and atypical depression: discussing environmental factors

This theme captures the perceived environmental factors of relevance to atypical depression. Most participants described the influence of socioeconomic factors on their symptoms. Specifically, financial pressure triggered a cycle of stress, maintained by their work environment and leading to continuously worsening mental health. For instance, one participant reported continuing to work although they felt unable to do so, due to income insecurity:

‘I used to walk around work and cry (…) I would force myself to go to work and I’d just be miserable at work, just be upset’. P13

Almost all participants also reported multiple, compounded social or environmental stressors around the time of atypical depression (‘there wasn’t one thing that set this off when I was younger’, P9*)*. Cyclical descriptions shared a pattern of feeling overwhelmed, unable to mitigate or cope with the onset of symptoms and, subsequently, leading to worse overall atypical depression:

‘It coincided also with the death of my father, and the breakdown of a marriage the previous year. So, it was like a flood of unpleasant things happening on personal and professional levels’. P6

Discussions also navigated to a unique factor in onset: National Health Service (NHS) waiting lists. The interviewer did not prompt this, nor was it present in the interview script. Rather, this naturally came out as being associated with both socioeconomic status and symptom course. There was a shared sense of helplessness (‘It almost feels like no one wants to help me, because no one wants to touch my case, which is very upsetting to me’, P5*).*

Waiting times carried a particularly complex meaning, particularly for individuals who had not responded to cognitive behavioural therapy (CBT) or who required access to specialised practitioners. This led participants to further fixate on social status, feeling that if the private sector was available to them, their atypical depression symptoms would be more manageable:

‘I feel like maybe if I would have had access to more money, then I would have been able to afford [therapy]. Then I could have reduced my symptoms (…) then maybe I would have known what to do the second time, and maybe the third time I wouldn’t have felt the way I felt’. P13

Individuals also described the cultural stigma behind the diagnosis of depression. This stigma contributed to some participants’ lack of peer support, which then clashed with their sense of self, causing a feeling of rejection from what is meant to be a core community. Consequently, stressors then clashed with their individual identity:

‘In the Black community, someone who is having mental issues gets overlooked (…) Black people are not supposed to be having issues with their mental health’. P3

### ‘Good and bad days’: atypical depression symptom variability

Discussions regarding day-to-day subjective experiences and triggers of atypical depression provided unique insight into the marked variability of atypical depression symptoms, which was portrayed as a frustrating experience where participants experienced ‘good’ days due to mood reactivity and ‘bad days’ due to fatigue (*‘*Everything feels a lot more energy draining … I’ll feel kind of spaced out’, P9). Participants exhibited heightened awareness around how the fluctuation of their atypical depression symptoms negatively affected them (‘It’s always kind of there … it kind of comes and goes in waves’, P12). This also applied to appetite fluctuations (‘Sometimes it’s overeating or not eating at all’, P4).

Changes in mood reactivity and behaviour habits were connected to heightened rejection sensitivity. This created a cycle of frustration, overeating, rumination and low self-esteem. In recurrent episodes, this pattern was viewed as enduring and internalised, with emotional fluctuation symbolising a significant change in participants’ beliefs and understanding of atypical depression:

‘I have a lot of support; I still really struggle. It almost seems like these symptoms are biological now (…) It just feels like now it’s something that’s intrinsic to me rather than to do with my environment’. P6

A subset of participants also experienced gradual progressions of their symptoms. Some participants described previously melancholic symptoms as ‘becoming’ atypical (‘I would say when I was a teenager, it was more typical symptoms of depression … I’d say that it’s atypical now’, P5). Participants described this transformation as a tangible shift, influencing how they experienced atypical depression over time (‘when I’m in that pit … the symptoms and everything that I experience, they do last’, P13). However, most participants continued to describe chronic or quick progressions of atypical depression episodes, with onset in early adolescence.

Descriptions of fatigue were universal in this sample, which seemed to oscillate from day to day. Feeling physically heavy, as well as physically and mentally exhausted, were emphasised as a primary impairment due to the visible effects in both physical appearances and environments (‘…I just felt like I had no energy to even make the effort to make people feel like I was doing OK’, P1*).* This visibility especially affected participants’ ability to successfully manage their symptoms, creating a challenging sense of uncertainty around their narrative understanding of atypical depression:

‘Today is a relatively good day and I’m feeling good. But a couple of days ago I was feeling dreadful (…) I hope that other people with depression have this (…) It’s not like a constant sadness, but it’s more often than not, but it’s so fluctuating at the moment it’s driving me nuts’. P9

### The search for coping mechanisms in atypical depression

Behaviours and other coping strategies participants used to manage their symptoms provided further insight into atypical depression aetiology and presentation. The majority reported not currently using medication, largely due to stigma or fears of dependence:

‘I don’t want to be taking pills and stuff like that because [laughs]; all my knowledge of it comes from movies and… they painted in a pretty bad light’. P10

For those describing prior medication use, they generally reported little or no response to multiple lines of treatment, which created a cyclical sense of frustration towards atypical depression:

‘I have felt quite frustrated with myself because I haven’t responded to the treatments I’ve tried. Or, I haven’t sort of responded enough’. P9

Although one participant described a positive response to medication (‘I did feel better on it. It helps, it’s a positive experience’, P3), most had sought psychosocial interventions through the NHS or third-sector services. CBT was the most commonly reported intervention, with overall positive or neutral results (‘CBT really helped my motivation’, P8), yet limited access or treatment response led many to adopt self-help strategies while awaiting further support (‘It’s been difficult to access treatment early, so I’ve taken up a little bit of self-help books’, P16).

Male participants particularly took pride in managing to prevent new episodes (‘I want to be able to do it for myself, overcome this and stand strong again’, P10*).* This desire for independence, however, also seemed to correlate to a desire to self-isolate. Perhaps to mitigate any potential impact of their symptoms on interpersonal relationships, male participants particularly seemed to discuss coping behaviours with a fear of being judged by their support network:

‘I started to say that I’m suffering with a lot of anxiety, I’m also very depressed. But even then, they were still saying (…) “oh you just need to do this and that”. I couldn’t get anybody to feel that emotional pain’. P16

Many participants reported that confiding in their support network was helpful. Engaging with their community through playing sports or spending quality time with their loved ones was denoted as particularly helpful and increasing resilience (‘I don’t think I would have been able to cope without that sort of communication’, P5). Generally, coping behaviours were focused on either physical or mental relaxation. Consistency and routine emerged as important grounding tools in the context of a lack of adequate access to mental health sources, but no coping mechanism was consistently effective:

*‘*What I’m trying to do is get back in my routine (…). I don’t know if it’s actually helping or it’s doing the opposite, but at least it keeps my mind distracted for certain periods of the day’. P10

## Discussion

This qualitative investigation sought to capture the lived experience of atypical depression presentations and perceived causes, including the role of gender-related influences. Similarly to previous literature, participants in this study described a key role of trauma exposure in atypical depression;^14^ however, our sample showed a clear awareness of the links among trauma exposure, atypical depression onset and increased comorbidity. This was then reported to be worsened by maladaptive responses to traumatic or adverse events, which created an interdependency between atypical depression episodes and increased interpersonal trauma. This has also been described in quantitative atypical depression literature.^12,14^

Participants characterised atypical depression as inextricably linked with anxiety symptoms and perceived considerable interdependence between the two. While increased comorbidity is characteristic of atypical depression, this has mainly been noted for bipolar spectrum disorder.^7,11^ However, around half of our participants reported either a diagnosed anxiety disorder or significant self-reported anxiety symptoms. While participants’ accounts resonate with literature linking these associations to shared genetic risk among internalising disorders,^11^ the complex interactions between genetic risks and familial environment seemed to hold particular significance in our participants’ explanatory models of atypical depression, similarly to other qualitative studies.^23,30^ While biological accounts offered diagnosis validation, social influences were universally regarded as more relevant to aetiology.^22^

Therefore, this study presents key insights into the role of patient beliefs: greater narrative understanding of atypical depression may award increased resilience, potentially highlighting the unique interactions between personal meaning and coping mechanisms in atypical depression.^23^ For instance, while one participant viewed caregiving as a stressor tied to gendered expectations, another found it fulfilling. This illustrates the relationship between participant beliefs and explanatory models; particularly for environmental factors, their effect on atypical depression was also defined by the meaning they held in participants’ narratives.

All participants in this study identified stress as a major contributing factor to atypical depression. Chronic stress was described as contributing to prolonged episodes, possibly reflecting lower stress tolerance shaped by trauma exposure.^12,14^ Work-related stress – also encapsulating financial and job dissatisfaction – was described as contributing to hopelessness, low mood and reduced self-esteem, similar to previous qualitative work.^22,30^ However, the present study’s findings regarding the relationships among stress reactivity, trauma response and atypical depression underscore how socioeconomic disadvantage and societal inequality represent a potential key influence on atypical depression. This warrants further examination in larger studies, suggesting the potential benefits of preventative interventions at the societal level.

This is additionally reflected in how limited access to psychological treatment represented a key barrier to recovery and a contributor to the chronicity of atypical depression for a significant number of participants. Long NHS waiting lists were described as both a stressor and a reflection of socioeconomic disadvantage. Specifically, waiting lists and inaccessible treatment within the NHS delayed access to early intervention, which often left participants feeling unable to make sense of, and manage, atypical depression onset.^31^ Without the financial means to ‘bypass’ these delays or access alternative support, some described feeling as though they were being ‘punished’ because of their socioeconomic status.^32^ This sense of exclusion echoes wider cultural conversations around therapeutic intervention accessibility, while also highlighting the emotional toll of feeling undeserving of care.^31^ The current sample also described feeling ‘guilty’ due to not responding to CBT or first-line medication. Internalised stigma surrounding treatment, particularly psychopharmacological treatment, also fuelled role conflict, characterised by conflicting pressures from incompatible expectations on an individual’s identity.^25,33^ Subsequently, participants engaged in alternative coping behaviours to avoid conflicts in their understanding of the self.

Gender-related factors were framed as influencing atypical depression onset, either as a primary driver or a secondary factor shaped by broader environmental stressors, through the lens of gender role conflict. Specifically, this exacerbated atypical depression onset while simultaneously affecting symptom presentation and help-seeking behaviour.^33^ Women often centred on pressure to maintain physical and social appearance, exacerbating rejection sensitivity.^34^ Additionally, menstruation represented a distinctly sex-related influence, shaping how participants’ understood episode progression and presentation. Womanhood was associated with stigma surrounding the physical effects of menstruation, reflecting the impact of ‘menstrual socialisation’: education around hiding and avoiding discussing menstruation.^35^ It is possible that the menstrual cycle may play a unique role for women in atypical depression, whether through social stress or hormonal interactions. Further investigation of this relationship appears justified.

Male participants’ views of depression as a weakness match previous studies.^33,36^ Gender role conflict was expressed as a personal failure tied to unmet provider expectations, which contributed to feelings of inadequacy and hopelessness.^33^ While this has been seen in men with MDD more generally,^36^ this study suggests that gender role conflict resolution was a key step in some atypical depression coping narratives: independently coping with atypical depression was seen as ‘redeeming’, resolving gender role conflict.^21^ Furthermore, men described more comfortably exhibiting help-seeking behaviour around women. This aligns with findings regarding the positive effect of embracing feminine traits in coping against psychological stress.^36^

These findings further resonate with the social role theory of sex differences, which conceptualises sex differences as stemming from social expectations attached to binary gender roles.^24^ Specifically, it argues that gender roles are socially produced through observations and reinforcement of ‘sex-typical’ behaviour, internalised as self-standards.^23,24^ Participants’ descriptions of caregiving obligations and hyper-awareness of negative consequences when deviating from gender role expectations can be understood through this lens. The distress associated with deviation from acceptable displays of atypical depression symptoms and coping mechanisms, described using gender-specific terms, reflects that the meaning attached to gender expression might have contributed to participants’ understanding of the perceived causes of atypical depression.^33,34^ Participants described the benefits and costs of adhering to gender roles; for example, both males and females in this sample expressed feeling strained by maintaining cultural ideals of femininity and masculinity during atypical depression episodes, which conflicted with symptom expression.

Among gender-diverse participants, gender-related stressors were noted as historically significant but not current drivers of atypical depression. Therefore, these findings indicate that gender-related conflict probably exacerbated other stressors and may not independently have caused atypical depression onset.^32^ Furthermore, both participants highlighted the protective role of peer support and self-compassion, aligning with findings on resilience in gender-diverse groups.

Overall, participants in this study showed considerable insight into the potential aetiological factors associated with atypical depression. All participants identified a mix of biological, environmental and gender- or sex-related contributions to atypical depression risk, onset or exacerbation. Trauma was particularly described as central to the narrative understanding of atypical depression aetiology, shaping perception and subsequent stress responses. Traumatic events were also discussed in relation to comorbidities that triggered or worsened atypical depression symptoms. Rather than a single aetiological influence, atypical depression onset was attributed to interactions of environmental, socioeconomic and gender-related stressors. Our findings thus highlight the importance of individual interpretations of perceived causes. Although no consistent explanation for the gender disparity in atypical depression emerged from participants’ accounts, these pointed to complex and variable combinations of social vulnerability, perceived genetic predispositions and environmental influences. Consequently, this study resonates with the need for clinicians and researchers to implement and recognise trauma-informed, gender-inclusive narratives in their practice. Considering the complexity in this sample’s explanatory models, these insights point to the need for such approaches to reduce the occurrence of individuals feeling overlooked or undervalued by current mental health and therapeutical frameworks.

The current study has potential clinical implications. Our sample described the possible origins of their atypical depression as comprising trauma, stress reactivity, interpersonal difficulties and identity-based stressors, which subsequently caused difficulties in both coping and treatment-seeking behaviours. Future studies would benefit from exploring the response of atypical depression to therapeutic approaches that go beyond symptom management. Trauma-focused CBT, dialectical behavioural therapy and psychodynamic psychotherapy may offer tailored support for emotional regulation and self-concept repair beyond traditional CBT. An exploration of this would be useful for primary care practitioners, because individuals with atypical depression described treatment-resistant illness, distinct symptom patterns and explanatory models. By recognising atypical depression as a diagnostic label and exploring tailored treatment options, this could significantly improve clinical outcomes and patient engagement for both primary and secondary care practitioners.

Furthermore, these findings have highlighted the importance of individual meaning assigned to aetiology. How individuals perceived stressors affected both resilience and explanatory narrative. This may be linked to the gender difference in atypical depression; gender role socialisation affected how individuals perceived stressors related to identity, further affecting vulnerability to atypical depression. However, this was not consistent across the sample, with varied emphasis placed on gender, socioeconomic status and cultural norms. While no definitive explanation for the gender disparity in atypical depression was identified, the relationship between gendered expectations and trauma warrants further exploration.^36^

### Limitations

The primary aim of the study was to investigate individual subjective perspectives on atypical depression aetiology, which we achieved through in-depth, semi-structured interviews. However, our sample consisted predominantly of participants in early to mid-adulthood; many were not engaged in caregiving responsibilities or in long-term, committed relationships, where gender-based effects tend to pervade.^27,33^ Therefore, subsequent qualitative work would benefit from a more diverse sample across both age and socioeconomic background. We acknowledge that there may have been some bias in sampling towards highly educated individuals in a Western, industrialised cultural context; this undoubtedly shaped the language surrounding, and conceptualisation of, perceived causes and stressors. Additionally, the sample contained only two gender-diverse individuals and therefore there was limited exploration of aetiological and gender-specific factors for this participant group in the current study. The current demographic was influenced by time constraints and the limitations of online recruitment; therefore, only a small number of gender-diverse individuals who consented to participation within the limited time window were included – we therefore acknowledge that their perspectives are underrepresented in the current analysis. Future research would benefit from targeted recruitment strategies within gender-diverse communities to allow for a thorough exploration of this subgroup’s experience with atypical depression; as well as via larger and more demographically diverse samples.

The online recruitment of participants and collection of data in this study meant that a high level of vigilance was required for the detection and exclusion of potentially invalid respondents, which may have been less critical in an in-person and/or clinical setting. Online recruitment may also have limited participation among individuals lacking reliable internet connection, digital literacy or access to video-call platforms, because the interviews required access to Microsoft Teams. Thus, it is acknowledged that this recruitment method may have also shaped the overall representativeness of the current sample. We also recognise that qualitative methods are not best suited for finding causal explanations, although our study has provided insights that might generate hypotheses for further quantitative research on this topic.

Finally, this study was conducted as part of a larger, mixed-methods project on aetiological factors in atypical depression, forming the basis of several postgraduate theses; therefore, there were significant time constraints affecting both recruitment and data analysis. Nonetheless, the lead analyst maintained a reflexive stance throughout all stages of the research process, with ongoing critical reflection to support the development of meaningful and well-supported themes rather than superficial interpretations.

## Supporting information

Toma et al. supplementary material Toma et al. supplementary material

## Data Availability

The data that support the findings of this study are available from the corresponding author (S.P.) upon reasonable request. The data cannot be made publicly available due to the inclusion of sensitive personal and medical information that would compromise the privacy of research participants. To request these data please contact the corresponding author.

## Supplementary material

The supplementary material is available online at https://doi.org/10.1192/bjo.2025.10924
