# Supplementary material for: Capturing subjective experiences of atypical depression: qualitative investigation of perceived aetiological factors and gender influences
Source: BJPsych Open. 2025 Dec 17;12(1):e19. doi: 10.1192/bjo.2025.10924 (PMC12724105; doi:10.1192/bjo.2025.10924)
Supplement: Toma et al. supplementary material [file S2056472425109241sup001.docx]

# **Supplementary Material: Semi-Structured Interview Guide**

Planned Interview Time: 45-60 minutes

Notes:

- Before beginning the interview, clarify that the interviewee does not have to answer all questions if they are uncomfortable.
- There may be contextual follow-up questions meant to clarify speaker intent and ensure the researcher does not subjectively interpret their experience to fit their research questions.
- Alternative questions in this interview guide may be asked if the participant does not respond or understand the initial question.

**QUESTION 1:** Can you tell me a bit about your experiences of depression symptoms?

**Prompts:**

- Which symptoms did you notice first?
- Did the symptoms develop quickly or slowly over time?
- Have the symptoms changed or stayed the same?

**QUESTION 2:** Do you have any sense of what might have caused the symptoms of depression?

**Prompts:**

- Were there specific life events that occurred in the 6-12 months before the mood symptoms started?
- In your view, were there particular biological, psychological or environmental factors that contributed to the development of the symptoms?

**QUESTION 2:** Did/do these symptoms affect your daily life?

**Prompts:**

- May I ask how?
- Did/do the symptoms affect your work/education, relationships, leisure activities, or any other area of your life?

**QUESTION 3**: Are there specific factors that seem to trigger your symptoms, or make them worse, on a day-to-day basis?

**Prompts:**

- For example, lifestyle factors or particular types of stressful situations?
- Was/is there something that helped you manage your symptoms?

**QUESTION 4:** Do you feel that your gender (identity) has influenced your experience with depression in any way?

- For example:
  - the factors that contributed to the symptoms
  - societal expectations of you
  - the roles you undertake
  - coping mechanisms
  - the way your symptoms are perceived and treated by others

QUESTION 5: Do you feel that your gender (identity) has influenced how you sought out support for depression?

**Prompts:**

- For example, did you feel that your gender (identity) affected how you could access support?
